# A Novel Intraurethral Device Diagnostic Index to Classify Bladder Outlet Obstruction in Men with Lower Urinary Tract Symptoms

**DOI:** 10.1155/2009/406012

**Published:** 2008-12-25

**Authors:** Leonardo O. Reis, Guilherme C. Barreiro, Alessandro Prudente, Cleide M. Silva, José W. M. Bassani, Carlos A. L. D'Ancona

**Affiliations:** Department of Urology, University of Campinas (UNICAMP), CEP 13083-970, Campinas, SP, Brazil

## Abstract

*Objectives*. Using a urethral device at the fossa navicularis, bladder pressure during voiding can be estimated by a minimal invasive technique. This study purposes a new diagnostic index for patients with lower urinary tract symptoms (LUTSs). *Methods*. Fifty one patients presenting with LUTSs were submitted to a conventional urodynamic and a minimal invasive study. The results obtained through the urethral device and invasive classic urodynamics were compared. The existing bladder outlet obstruction index (BOOI) equation that classifies men with LUTSs was modified to allow minimal invasive measurement of isovolumetric bladder pressure in place of detrusor pressure at maximum urine flow. Accuracy of the new equation for classifying obstruction was then tested in this group of men. *Results*. The modified equation identified men with obstruction with a positive predictive value of 68% and a negative predictive value of 70%, with an overall accuracy of 70%. *Conclusions*. The proposed equation can accurately classify over 70% of men without resorting to invasive pressure flow studies. We must now evaluate the usefulness of this classification for the surgical treatment of men with LUTSs.

## 1. Introduction

Invasive pressure flow studies (PFSs) in urodynamics are still the gold standard method for objective classification of bladder outlet obstruction (BOO) in men with lower urinary tract symptoms (LUTSs). It is able to delineate patients for successful surgical approach [1], however, it is still costly, time-consuming, and associated with significant morbidity.

The risks of complications after conventional urodynamic study in men with BOO are greater, and acute urinary retention, macroscopic hematuria, urinary tract infection, and/or fever can occur in over 19% of the cases [2].

During the past 14 years, many experts have raised minimal invasive possibilities as substitutes [3, 4].

Griffiths et al. have previously described and validated a minimal invasive technique based on controlled inflation of a penile cuff during voiding [5]. Others had attempted other types of penile cuffs and condoms with insufficient results [3, 6–9].

Trying to overcome these limitations, we developed, in association with the University's Biomedical Engineering Department, a urethral device capable of extracting from the voiding patient measurements comparable to those achieved from invasive PFS [10].

We have published before that applying logistic regression fitting to the minimal invasive method utilizing this urethral device was able to detect most patients with BOO [10]. However, the conventional urodynamic assessment to be avoided, an equation as simple as Abrams-Griffiths number [11] is necessary.

## 2. Material and Methods

The bladder outlet obstruction index (BOOI) was created to classify men as obstructed, equivocal, or unobstructed based on invasive urodynamic results of *p*_det_ at *Q*_max_ and *Q*_max_ itself [12, 13].

These were plotted on an equation and the BOOI obtained could differentiate the three groups by a nomogram analysis (ICS nomogram) [11, 14, 15]:

(1) $$\text{BOOI}\;=\;(p_{\det\;}\;\;\text{at}\;\;Q_{\max\;})\;-\;(2\;\times \;Q_{\max\;}).$$

Once logistic regression analysis has demonstrated 67% sensitivity and 79% specificity utilizing the novel intraurethral device to identify BOO [10], we developed a new equation from the comparison of the mathematical relationship between invasive *p*_det_ at *Q*_max_ and *Q*_max_, and noninvasive *p*_iso_ and *Q*_interr_ results, respectively. Therefore, we included in the mathematical comparison all the new variables that the urethral device test introduced to the classical invasive measures.

### 2.1. Patients

After obtaining the Ethics Committee approval and a written informed consent, sequential invasive and minimal invasive PFSs were prospectively performed in men with clinical complaints of LUTS.

### 2.2. Invasive PFSs and Gold Standard Classification of Obstruction

PFS was performed according to ICS good urodynamic practice guidelines [16], with the patients in the standing position. A 6Fr double lumen urethral catheter was inserted for filling and bladder pressure measurement, and a 6Fr rectal pressure line was inserted for abdominal pressure measurement. External transducers were leveled with the pubic symphysis and zeroed to atmospheric pressure. The bladder was filled with physiological saline at 50 mL\minute until maximum cystometric capacity. Flow was recorded by a load cell. The *p*_det_ at *Q*_max_ and *Q*_max_ was measured on a computer display, using a cross-wire cursor and plotted on the ICS nomogram [11] to classify obstruction.

### 2.3. Minimal Invasive Urethral Device Test

**Figure 1:**
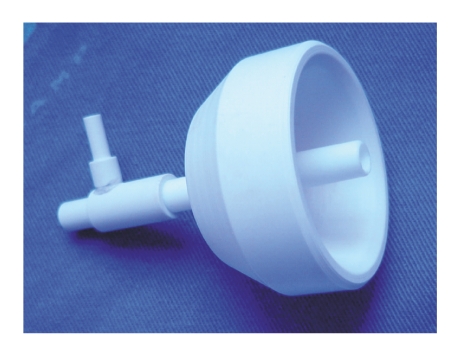
Actual urethral device used in the noninvasive test.

The minimal invasive urodynamic evaluation was done using a urethral device [10] especially designed in conical shape to be adapted to the urethral meatus and fossa navicularis, with a side opening to connect the pressure transducer and the other end free for the release of urine/saline solution (Figure 1). The internal diameter of the device is 4 mm, and the lower external diameter is 6 mm. It consists of polyvinyl, carbon, and polytetrafluoroethylene, making it light, non-distensible, and easy-to-sterilize.

After concluding the conventional urodynamic assessment, the postvoid residual urine volume was measured. The bladder was refilled with warm saline solution (37°C) at the same maximal cystometric capacity, and the 6F urethral catheter was removed. This study was not blinded. The urodynamic equipment used to register the conventional and minimal invasive studies was the Dantec, Minuet Compact model.

During voiding, the patient was instructed to interrupt the flow with a digital maneuver that simply blocked the end of the urethral device. Interrupted urine flow (Qinter) and isometric bladder pressure (Piso) were registered using this technique. The rectal catheter used in the conventional urodynamic assessment was maintained to record the Pabd. The Piso was measured at the greatest pressure point after interrupted flow, and the Qinter was considered the plateau: the greatest flow point resulted from the impact of urine in the flow meter and was considered as interference [17, 18].

### 2.4. Analysis

These variables (Piso and Qinter) were compared using the Pearson's coefficient correlation test [19, 20] to their correspondent measures obtained through invasive urodynamics, which were *p*_det_ at *Q*_max_ and *Q*_max_, respectively.

As only two variables demonstrated predictive ability in a previous analysis including all variables (isometric pressure, abdominal pressure, maximal flow, Qinter, and postvoid residual urine volume) [10], a binary result of obstructed versus nonobstructed, obtained by grouping the Abrams-Griffiths classification, was done. The normal and equivocal categories were combined as the nonobstructed group.

We tried to find a function of minimal invasive data to approximate (i.e., to classify), as closely as possible, the reference standard classification. This mathematical correlation was tested to determine substitute equivalents for an adapted Abrams-Griffiths equation worth for minimal invasive measures.

A linear correlation between the minimal invasive *Q*_interr_ and invasive *Q*_max_ as well as a quadratic relationship between the pressures *p*_iso_ and *p*_det_ at *Q*_max_ was demonstrated plotting on the graph the correspondent variables. The numeric translation for these relations is illustrated as follows:

(2) $$Q_{\max\;}\;=\;a\;+\;(b\;\times \;Q_{\text{interr}}),$$

(3) $$p_{\det\;}\;\;\text{at}\;\;Q_{\max\;}\;=\;a^{\prime }\;+\;(b^{\prime }\;\times \;p_{\text{iso}})\;+\;(c^{\prime }\;\times \;p_{\text{iso}}^{2}).$$

The *a* and *b* parameters are estimated from the simple linear regression on (2); and *a*′, *b*′ and *c*′ are parameters estimated from the multiple linear regression on (3).

When we substitute (2) and (3) into the original equation of Abrams-Griffiths for invasive urodynamics (1), a new function to determine urinary outlet obstruction through minimal invasive measures is described. The values obtained using the urethral device test can be plotted on this new equation. The final result was equivalent to the Abrams-Griffiths parameters for classification upon invasive test, despite using minimal invasive measures. This equation was able to distinguish the individuals into two different groups: obstructed, equivocal/unobstructed. The sensitivity, specificity, positive predictive value (PPV), and negative predictive value (NPV) were calculated for the minimal invasive results in comparison to their invasive classification.

All computations were done with Statistical Analysis Systems (SAS Institute, Cary, NC), version 8.2 [21].

## 3. Results

### 3.1. Patients

Fifty-one consecutive male patients with complaints of lower urinary tract symptoms were included in this study. Among these men, 46 (90%) were suitable for analysis, 5 (10%) were excluded due to involuntary high-amplitude detrusor contractions and low bladder compliance during the invasive test.

The mean age was 64.8 ± 8.5 years (30 to 82). The prostate weight by digital rectal examination was of 39.2 ± 18.8 g.

The mean international prostatic symptom score (IPSS) was 14 ± 6.9. The mean value for the normal patients (8, range: 1–12) was lower than those of the equivocal patients (13, range: 1–22) or obstructed patients (13, range: 4–23). However, the results of the analysis of variance were not significant (*P* = .14).

The mean postvoid residual urine volume was 48.9 mL (range: 0–250). The mean values of the normal patients (45 mL, range: 0–110) were lower than those of the equivocal patients (60 mL, range: 0–250) or obstructed patients (65 mL, range: 0–140). The comparison of the mean postvoid residual urine volume between the groups with the different urodynamic diagnoses was not statistically significant (*P* = .96).

The urethral devices used in the minimal invasive urodynamic evaluation did not cause pain during the procedure. Leakage occurred between the urethra and the device in 1 patient, and the examination was repeated.

### 3.2. Invasive Classification

Using invasive data, 21 (45.6%) were classified as obstructed, 15 (32.6%) as equivocal, and 10 (21.7%) as unobstructed; a total of 25 (54.4%) of equivocal/unobstructed. When Pabd was added to the statistical analysis, no additional patient was identified as obstructed.

### 3.3. Minimal Invasive Results and Classification

Significant linear correlation was observed between invasive *Q*_max_ and minimal invasive *Q*_interr_, *r* = 0.558, *P* < .0001, and a quadratic polynomial correlation was observed between invasive *p*_det_ at *Q*_max_ and minimal invasive *p*_iso_. Thus, through simple linear regression for the urinary flow values and multiple linear regression for the pressure values, we found the numeric equivalents that substitute the variables in (2) and (3), as follows:

(4) $$Q_{\max\;}\;=\;a\;+\;(b\;\times \;Q_{\text{interr}}),\;\text{where}\;a\;=\;2.007,\;b\;=\;0.627,$$

then,

(5) $$\begin{array}{c} Q_{\max\;}\;=\;2.007\;+\;(0.627\;\times \;Q_{\text{interr}}),\\ \begin{array}{c} p_{\det\;}\;\;\text{at}\;\;Q_{\max\;}\;=\;a^{\prime }\;+\;(b^{\prime }\;\times \;p_{\text{iso}})\;+\;(c^{\prime }\;\times \;p_{\text{iso}}^{2}),\\ \text{where}\;a^{\prime }\;=\;72.722,\;b^{\prime }\;=\;-0.679,\;c^{\prime }\;=\;0.004, \end{array} \end{array}$$

therefore,

(6) $$\begin{array}{c} p_{\det\;}\;\;\text{at}\;\;Q_{\max\;}\;=\;72.722\;+\;(-0.679\times p_{\text{iso}})\;+\;(0.004\times p_{\text{iso}}^{2}). \end{array}$$

This allowed for a mathematical substitution of values on the Abrams-Griffiths original equation (1) [12] for the numeric nonivasive correspondents achieved through these comparisons. Through equivalent substitution, we reached

(7) $$\begin{array}{ll} \text{BOOI} & =(p_{\det\;}\;\;\text{at}\;\;Q_{\max\;})-(2\times Q_{\max\;}),\\ \text{BOOI} & =\{72.722\;+\;(-0.679\times p_{\text{iso}})+(0.004\times p_{\text{iso}}^{2})\}\\ & \;-2\times \{2.007\;+\;(0.627\times Q_{\text{interr}})\},\\ \text{BOOI} & =68.708-0.679\times p_{\text{iso}}\;+\;0.004\times p_{\text{iso}}^{2}\\ & \;-1.245\times Q_{\text{interr}}. \end{array}$$

This was the final equation for classification of BOO using the urethral device test, and the result was denominated urethral device number (UD_*n*_):

(8) $${\text{UD}}_{n}\;=\;68.8\;-\;0.68\;\times \;p_{\text{iso}}\;+\;0.004\;\times \;p_{\text{iso}}^{2}\;-\;1.25\;\times \;Q_{\text{interr}}.$$

The final result (UD_*n*_) classified each patient as obstructed or equivocal/unobstructed, according to the ICS nomogram [11], which was not modified.

Of the 21 men classified as obstructed by Abrams-Griffiths equation (conventional invasive urodynamic), 13 were identified by the minimal invasive diagnostic index (Table 1).

This new equation classified 27 (58.7%) of the patients as equivocal/unobstructed and 19 (41.3%) as obstructed. Sensitivity was 61.9%; specificity, 76%; PPV, 68.4%; NPV, 70.37%. Overall accuracy was 69.6% (Table 2).

### 3.4. Methodology Comparison

**Figure 2:**
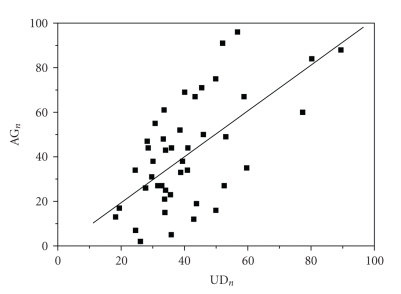
Linear correlation tested through the Pearson's coefficient between the number of Abrams-Griffiths (BOOI: *y* axis) and the urethral device number (UD_*n*_: *x* axis), *r* = 0.653, *P* < .0001.

In order to prove methodological equivalency, we tested the Pearson's coefficient correlation [19, 20] on the final results. The graphic correlation between the numeric BOOI and UD_*n*_ for each patient is demonstrated on Figure 2, *r* = 0.653, *P* < .0001.

## 4. Comment

This idea was original and the device was designed in our institution; therefore, this is the first report of this method.

The analysis of the IPSS of the patients in this study did not identify patients with BOO. However, none of the patients presented with an IPSS of 28 or greater, a score that has a positive correlation with obstruction [22].

In this study, no correlation was found between the postvoid residual urine volume and BOO. Other studies have also failed to demonstrate this association [23, 24].

The probability of a urinary infection from the use of the urethral device is low because it is introduced only up to the fossa navicularis, and it does not cause pain.

Other techniques of noninvasive urodynamic evaluation have reported problems such as elasticity in the condom catheter. Also, different types of material and different sizes of the penile cuff can register a greater isometric pressure [25, 26]. These inconveniences were not observed with the urethral device although we considered this technique minimal invasive.

Because the minimal invasive assessment was performed immediately after the conventional assessment, a low variability was warranted as demonstrated before in the second pressure/flow study of 192 patients with the diagnosis of BOO maintained in 95.2% and reduced only 6.9% in detrusor pressure at maximal urinary flow [27]. This variation, although slight, was probably due to the reduced urethral resistance resulting from successive voiding within a short period.

The results obtained revealed 67% sensitivity and 79% specificity, applying a logistic regression [10], and 62% sensitivity and 76% specificity applying the new equation fitting to the minimal invasive method utilizing this urethral device to diagnose BOO.

These results are similar to those reported in other studies, in which the condom catheter noninvasive urodynamic assessment was used to correctly diagnose 77% of the patients who presented with obstruction [6]. It is important to note that 25.3% of the patients in that study were excluded from the analysis because of problems such as urine loss between the condom and penis (13 patients), faulty records (3 patients), discomfort (2 patients), and the inability to urinate (1 patient). None of the patients using the urethral device were excluded because of technical problems.

The noninvasive urodynamic study using a cuff also helped to separate the obstructed from the unobstructed patients. The sensitivity and specificity rates for the diagnosis of BOO in a study of 116 patients were 73% and 75%, respectively [28]. The penile cuff is inconvenient, because it makes voiding impossible for some patients, provokes urethral bleeding, and increases the isometric bladder pressure [8].

Griffiths et al. obtained comparable results, which were actually useful for only two thirds of the patients previously considered suitable for this method (i.e., two thirds of 54% from the total recruited men). This limited result could probably be due to the method itself, which did not differ a lot from the simple flow rate criterion, in addition to the inconvenience of wearing a penile cuff, which restricted the overall evaluation [5].

The condom catheter and the penile cuff are also not effective in identifying normal and equivocal patients [29]. This suggests that a follow-up examination is necessary for these patients and, depending on the outcome, a conventional urodynamic assessment should be conducted.

Differently from what has been done so far [5], our hypothesis was that changes in the original equation could adapt minimal invasive results, obtained with the urethral device, to the ICS nomogram. The results would be eligible for classification on this nomogram, which was not modified. The subsequent prospective study demonstrated accordance between the proposed minimal invasive and invasive classifications.

Measures obtained through the urethral device were comparable to the invasive correspondents [10] and enabled the modification of the Abram-Griffiths original equation [12]. The new formula can be applied to classify any other individual submitted to the urethral device test.

Taking into account that data were obtained through different, though comparable, methods, it was necessary to adequate parameters to use the standard Abrams-Griffiths equation. The values of pressure and urinary flow measured minimal invasive ultimately correlated to the invasive measures. This happens because the minimal invasive method suffers external influences, such as urethral compliance and abdominal pressure, which do not primarily affect the direct measures achieved through invasive urodynamics [14, 15]. In spite of using the same equation as a basis for minimal invasive classification of BOO, there was a mathematical grant, since the errors observed between the two methods were constant. This allowed for an equivalence represented by the new equation (8).

When we apply the minimal invasive diagnostic index (UD_*n*_) to the 50 patients analyzed in our previous study [10], the same 70% accuracy was confirmed, showing this method validation.

## 5. Conclusions

The urethral device test proved to be a promising substitute for invasive evaluation of men presenting with LUTS after at least two different methodologies and more than a hundred patients enrolled with over 70% accuracy [10].

Primary results presented good correlation to the gold standard method, even though there is still much to improve. It is an easily performed, acceptable method located between the free simple flow rate criterion and PFS, and may represent a reasonable option for BOO diagnosis in the near future.

These preliminary results need a greater number of men evaluated and the definition of a final clinical use for its classification. It has not yet been proven if the results could correlate with a good outcome after transurethral prostatectomy. A prospective clinical study is under way to assess this new method in relation to the outcome of elective prostatectomy [30, 31].

## Figures and Tables

**Table 1:** Comparison between Abrams-Griffiths equation (conventional invasive urodynamic) and minimal invasive diagnostic index.

	Minimal invasive diagnostic index	
Conventional urodynamic	Normal and equivocal	Obstructed
Normal and equivocal	19	06
Obstructed	08	13

**Table 2:** Measurements of accuracy for obstruction through the new method.

Measurement	%	95% CI	*n*/total
Sensitivity	61.9	38.7; 81.1	19/21
Specificity	76.0	54.5; 89.8	19/25
Positive predictive value	68.4	43.5; 86.4	13/19
Negative predictive value	70.4	49.7; 85.5	19/27
Accuracy	69.6	54.1; 81.8	32/46

CI: confidence interval, *n*: number of patients.
